# The opinions and experiences of nurses on frailty screening among older hospitalized patients. An exploratory study

**DOI:** 10.1186/s12877-021-02586-z

**Published:** 2021-11-03

**Authors:** Ron M. J. Warnier, Erik van Rossum, Monique F. M. T. Du Moulin, Marjolein van Lottum, Jos M. G. A. Schols, Gertrudis I. J. M. Kempen

**Affiliations:** 1 Care and Public Health Research Institute (CAPHRI), Department of Health Services Research, Maastricht University, Maastricht, The Netherlands; 2 Envida, Care for Elderly, Department of Treatment and Guidance, Vijverdalseweg 10, 6226 NB Maastricht, The Netherlands; 3 Academy of Nursing, Zuyd University of Applied Sciences, Heerlen, The Netherlands; 4 Care and Public Health Research Institute (CAPHRI), Department of Family Medicine, Maastricht University, Maastricht, The Netherlands

**Keywords:** Frailty screening, Nurses opinions, Hospitalized older patients, Frailty

## Abstract

**Background:**

Routine screening for frailty at admission by nurses may be useful to detect geriatric risks and problems at an early stage. However, the added value of this screening is not clear yet. Information about the opinions and attitudes of nurses towards this screening is also lacking. As they have a crucial role in conducting this screening, an exploratory study was performed to examine hospital nurses’ opinions and perspectives about this screening and how it influences their daily work.

**Methods:**

A qualitative, exploratory approach was employed, using semi-structured interviews with 13 nurses working on different general medical wards (surgical and internal medicine) in three Dutch hospitals. Frailty screening had been implemented for several years in these hospitals.

**Results:**

The participating nurses reported that frailty screening can be useful to structure their work, create more awareness of frail older patients and as starting point for pro-active nursing care. At the same time, they assess their clinical view as more important than the results of a standard screening tool. The nurses hardly used the overall screening scores, but were particularly interested in information regarding specific items, such as delirium or fall risk. Screening results are partly embedded systematically and in daily nursing care, e.g., in team briefings or during transfer of patients to other wards. The majority of the nurses had received little training about the background of frailty screening and the use of screening tools.

**Conclusions:**

Most nurses stated that frailty screening tools are helpful in daily practice. However, nurses did not use the frailty screening tools in the referred way; tools were particularly used to evaluate patients on separate items of the tool instead of the summative score of the tool. When frailty screening tools are implemented in daily practice, training needs to be focused on. Additional research in this field is necessary to gain more insight into nurses’ opinions on frailty screening.

**Supplementary Information:**

The online version contains supplementary material available at 10.1186/s12877-021-02586-z.

## Background

Due to our ageing society, more older patients will be admitted to acute hospitals in the near future. Nowadays, in the Netherlands, approximately one third of admitted hospital patients are aged 70 years and over [1]. As a result, acute care hospitals are becoming more and more geriatric services, approximately one third of the admitted patients is 70 years and over [2]. This affects the work of the nursing staff are responsible for the care of these older patients at all times [3].

Acute hospital admissions are not without risks for older patients. They are associated with an increased risk of negative health outcomes such as iatrogenic complications, delirium, and functional decline [4–6]. These risks are even higher in frail older patients. Approximately 30–60% of hospitalized older people lose the ability to perform relevant activities of daily living, compared with their pre-admission level of functioning [7, 8]. Andela and colleagues reported that 50–80% of older hospitalized patients are considered frail [9]. Functional decline and frailty contribute to negative short and long-term health outcomes [10], such as a prolonged hospital stay [11], frequent readmission to hospital, admission to a nursing home, and increased mortality [12, 13]. Frail patients have a higher risk for functional decline compared with their nonfrail counterparts (RR 1.32). Frail patients have a relative risk for in-hospital mortality and mortality in medium- and long-term compared to nonfrail (in-hospital RR: 8.20, medium RR: 9.49 and long RR: 7.94). The overall mortality risk in frail individuals is 3.49 times compared to nonfrail, respectively. Length of hospital stay was higher for frail older adults (13.5 days) compared with nonfrail (8.3 days) [14, 15].

During acute admission, routine care focuses particularly on diagnostic and therapeutic interventions, while general geriatric problems (e.g. cognitive impairment and functional decline) are often overlooked and seem to be relatively unrecognized [16]. Parke and colleagues suggested that early detection of geriatric risks and problems can improve functional outcomes in these patients [3]. Early identification of patients at risk can help nurses to start preventive care in combination with good basic care and additional tailored geriatric care [4, 17]. Multidimensional interventions on physical, nutritional, psychological and social domains are effective and can prevent negative health outcomes [18]. Screening results can be seen as a starting point for comprehensive geriatric assessment (CGA) an can also support decisions for CGA [19]. In most Dutch hospitals, systematic screening on frailty is performed by nurses at hospital admission [20], for which different screening tools are used.

Many studies have been published on the psychometric properties of different frailty screening tools for use in hospitals [21]. Data about their applicability, feasibility and usefulness is scarce and there is still debate on the added value of these screening tools [21]. Also, hardly any information is available yet about the opinions and attitudes of nurses towards systematic and standardized frailty screening in both community and hospital settings. These data are important because nurses are often the professionals who conduct the screening, assess the screening data and initiate preventive interventions based on screening outcomes.

Coker and colleagues explored the view of care staff on frailty and frailty screening in community dwelling older persons [22]. Nurses stated that multi-domain frailty screening (e.g., on physical, mental health and psychological, social, environmental, and economic factors) is necessary to support interdisciplinary working for older patients in the community. They mentioned that, in addition to observing and asking questions, screening tools are necessary to assess frailty. Nurses stated that an optimal frailty tool would help them to understand frailty in its different domains. On the other hand, they considered that more training was needed on the understanding of frailty in general and the use of frailty screening tools specifically [22]. Nursing perceptions on screening tools for delirium risk were studied in a clinical palliative setting [23]. Nurses stated here that screening tools could support the documentation of observations of patient symptoms. Although delirium is a common problem in palliative care, screening tools were not routinely used to ensure early recognition by nurses. Nurses mentioned that screening is only one step in the complex care for delirious patients and that the follow-up step (initiating preventive and pro-active care) is even more important than the screening tool itself.

As information on frailty screening by nurses in acute hospital practice is scarce, so we conducted an explorative study to examine their opinions on frailty screening and how it impacts their daily work. These findings may contribute to optimizing pro-active care for older frail hospitalized patients.

## Methods

### Design

We used an exploratory qualitative approach using semi-structured interviews with nurses working on different wards in acute hospitals.

### Setting

Nurses working in three hospitals in the south of The Netherlands were included in the study: one moderate sized general hospital (hospital 1, about 300 beds), one large general hospital (hospital 2, 600 beds) and one large university hospital (hospital 3, over 700 beds). In all three hospitals, initial screening by nurses during admission for frailty had been implemented for several years and older patients were admitted to almost all wards throughout the hospital. We did not include hospital sites with specific geriatric wards as a broader geriatric knowledge and approach in such wards could bias our findings. Nurses on geriatric wards receive additional geriatric training, whereas we were primarily interested in the opinions of general nurses.

### Screening for frailty in the included hospitals

In hospital 1, all older patients are screened at admission by means of the 13-item RISK scale. This scale was developed by the geriatric team of the hospital based on the Dutch National Safety Management Program for the screening of frail hospitalized older patients (in Dutch abbreviated as “VMS”). The RISK scale has not been validated yet and is integrated in the nursing assessment. The screening consists of four domains: delirium risk (three questions), fall risk (one question), malnutrition (three questions) and risk of functional decline (six questions) [20]. Geriatric consultation by a specialized geriatric nurse is provided when frailty is identified (a score ≥ 4 on the RISK scale).

All admitted older patients In hospital 2 are screened by means of the validated 15-item Groningen Frailty Indicator (GFI) [24]. The GFI consists of four domains: physical (nine items), cognitive (one item), social (three items) and psychological (two items). The items of the screening tool are fully integrated in the nursing assessment, which is assessed during admission. Based on the outcome of the GFI assessment (score ≥ 4), members of the specialized geriatric team first check the patient file and decide upon whether a further geriatric assessment of the patient is necessary.

In hospital 3, all admitted patients of 70 years and over are screened by means of the validated Maastricht Frailty Screening Tool for Hospitalized Patients (MFST-HP) [25]. This 15-item tool consists of three domains: physical (nine items), psychological (four items) and social (two items). The MFST-HP is fully integrated in the initial nursing assessment, and afterwards the digital system generates a frailty score. Digital standardized nursing protocols for each of the MFST-HP items are available within the nursing system. A patient is labelled as frail when the MFST-HP score is 5 or higher [2]. A specialized geriatric team pro-actively visits all patients with a specific cut-off score on the frailty screening for further comprehensive geriatric assessment.

### Participants

Nurses were included in the study if they were employed at general wards (internal or surgery wards) in each of the selected hospitals; many older patients are admitted to particularly these wards. Nurses working on ‘high-care departments’ such as emergency departments or intensive care units or nurses working on a geriatric ward were excluded from the study. A variety of nurses according to gender, age and educational level (i.e., secondary vocational education or higher professional education) was recruited for this study by geriatric nurse practitioners of the included hospitals by means of purposive sampling. In view of the qualitative approach, we limited recruitment to approximately four to five nurses per hospital. After selection, an information letter on the study and content of the interview was handed over to the participants. Informed consent was provided by all participants. Interviews were conducted at the hospital sites.

### Data collection

Nurses were interviewed in a semi-structured way by two members of the research team (authors RMJW and MvL). The interviews in hospital 1 were conducted with individual nurses (*n* = 4), the interviews in hospital 2 and 3 were conducted in small groups: one individual interview, two pairs, and one group of four nurses. Differences in group composition was due to practical issues. In total, eight individual or group interviews were conducted (i1 t/m i8). Author MvL served as moderator of all interviews, author RMJW observed the interviews and made additional notes. An interview guide was constructed based on literature and expert consultation and pre-tested in a hospital ward that was not included in the present study (see supplementary files). No major adjustments of the interview guide were necessary. Nurses in all hospitals were asked the same series of open-ended questions regarding two main topics related to our research question. The first topic was about the nurses’ opinions of screening and screening tools in general of frailty screening among older patients, the second topic was about the nurses’ perceptions on the impact of the frailty screening on daily nursing practice. Based on the responses in-depth follow-up questions were asked to further clarify and expand on areas that seemed to be of interest or concern of the participants. All interviews were recorded. At the end of the interview the researcher summarized the findings of the interview as a part of member checking. Transcriptions were checked by the participating nurses. Socio-demographic and background data such as age, gender and educational level were collected at the end of the interview.

### Data analysis

All interviews were transcribed verbatim by two members of the research team (authors RMJW and MvL) and two authors (MFMTD and RMJW) analysed the data via qualitative content analysis using open coding [26] . They were both blinded for each other’s coding and initial codes were discussed afterwards. In the second step codes were edited via axial coding; some codes were divided, some other were combined. In the last step selective coding was used to combine different issues. Illustrative quotations for specific opinions were selected by authors MFMTD and RMJW and were labelled with a specific code to ensure anonymity. Pair and group interviews were labelled as one unit of interview. The COREQ checklist was used to report the data [27] .

## Results

All 13 invited registered nurses in the three hospitals agreed to participate. Their ages varied from 21 to 63 years, and ten of them were female (see Table 1). Their experience as a registered nurse in hospital care ranged from less than 1 year to 45 years. Four nurses indicated that they had no specific experience in nursing older patients, but all others did have this experience, varying between 6 and 45 years. The nurses had different levels of education: six nurses graduated secondary vocational education while seven had a bachelor’s degree. The majority of the nurses [10] had no specific geriatric education, while two had received specific training such as clinical lessons from a geriatrician or an e-learning program on geriatric care.

**Table 1:** Characteristics of the sample

Female (%)	10 (77)
**Age (SD)**	**38 (SD 11.8; range 21–61)**
**Highest education level (%)**	
**Secondary vocational education (%)**	**7 (54)**
**Higher professional (nurse) education (%)**	**6 (46)**
**Course in geriatric care (%)**	**3 (23)**
**Years of experience as a nurse, mean (SD)**	**17 (13.0; range 2–45)**
**Hospital experience, mean (SD)**	**17 (12.8; range 2–45)**
**Full time employment (%)**	**13 (100)**
**Experience taking care of older patients (SD)**	**11.6 (SD 14.1; range 0–45)**

*SD* standard deviation

After the first analyses and discussion between the two authors MFMTD and RMJW, 15 initial codes were extracted from the interview data. These codes were then renamed or combined by both authors, resulting in two main themes corresponding with the interview guide: (1) the nurses’ (general) opinion about screening and (2) the experiences and impact of screening on their daily work. Finally, six subthemes were derived from these two main themes. Four of them relate to the nurses’ opinions: (1.1) nurses’ attitudes towards frailty screening; (1.2) the importance of their clinical view versus the outcomes of screening tools; (1.3) advantages and disadvantages of frailty screening in general; (1.4) prerequisites and suggestions for improvement. The other two subthemes relate to their experiences: (2.5) the use of and knowledge about frailty screening in daily care and briefings within and between teams; and (2.6) follow-up actions after screening.

### Nurses’ opinions about screening (subthemes 1.1 to 1.4)

#### 1.1: attitude towards screening

In two hospitals nurses stated that frailty screening is part of their job*: “It’s just part of our job. It is part of it just like pain assessments for example*” [i1]. Some nurses thought that the use of a certain screening tool could be helpful. “*It* (use of screening, authors) *must increase your alertness, especially for frail patients, and also* generate *a to-do list for us which actions have to be taken. That’s why these screening tools are created, I think”* [i2]. Some experienced nurses stated that screening tools in general could be helpful for younger (and less experienced) nurses. One said*: “If you are just starting out in the nursing profession, it is sometimes helpful to have some tools”* [i3].

#### 1.2: clinical view versus screening results

All nurses stated that their clinical view and professional observations are more important than the overall summative score of a frailty screening tool. In one hospital, a nurse said that she always added her observations to the frailty scores. Two nurses [i5, i4] stated that the outcome of the frailty screening tool was used to substantiate or to confirm their clinical view. Another nurse mentioned that she felt that screening is sometimes considered more important than her own knowledge and expertise with respect to taking care for older adults: *“I sometimes feel that a score on the list is considered as expertise on itself by others, and I think that it has to be perceived as an aid, and not as a strict guide”* [i4]. Most nurses stated that the outcomes of a screening tool cannot replace the clinical expertise and view of the nurse. Two nurses on a surgical ward stated: *“Let me put it this way: when I get an overview of the patients on the screen, I cannot see who is a vulnerable older person. I can see whether someone should be resuscitated or not, but there is no indication of a colour or a certain sign that the patient is a vulnerable older person. I have to visit the patient at his bed for to see if he is vulnerable, huh …*” [i4]. Another nurse stated that the screening tool could be complementary for her as a professional: *“Sometimes an assessment instrument is very helpful, but hey, I think it must always be combined with what you see and what you observe as a nurse. A score cannot express this. So yes, it is helpful”* [i3]. Most nurses mentioned that the score on the individual items of the screening tool could be more helpful than the summative total frailty score. The individual items could help nurses with focusing on several geriatric items as for instance delirium, fall risk or malnutrition [i2].

#### 1.3: advantages and disadvantages of screening

All nurses stated that due to screening nursing interventions in general were started more pro-actively at an earlier stage after admission. Furthermore, screening and screening results can create more awareness for potential risks when older patients are admitted to the hospital. One nurse stated*:” Every time when I screen older patients, the screening triggers me again and again*” [i6]. Another one mentioned:” *It is surely a helpful tool, and it is also good that the screening is mandatory to complete. I think if it had been a separate form, it would be easier to ignore it, but now you cannot ignore it. So, it is definitely a tool that assesses the physical and psychological functioning of the patient at admission”* [i2]. Screening can help nurses to visualize the older patients’ risks or problems when admitted according to two nurses. Two other nurses stated that, due to early interventions on potential risks, the tool was considered as a kind of preventive tool. Screening can therefore substantiate the clinical view of nurses. The tools can help nurses to structure their work in taking care of older patients. One of them stated: *“For example, if you are reading the patients’ file and you do not know the patient at all* (after some days off duty, authors)*, then you must have actually visited the patient to know the patient’s level of functioning; in that case the items of the screening tool could be helpful as a sort of overview”* [i2]. Nurses stated that using the tool may help to create uniform structure in patient reports within nursing teams and could be helpful to monitor the patients functioning during the hospital stay.

None of the interviewed nurses reported specific disadvantages of frailty screening as such. However, they perceived some disadvantages of the used screening procedure. One nurse mentioned that, due to automatically generated screening results and follow up actions (i.e., automatic generated consultation request for the geriatric team) in the background of the digital patient file, the actual frailty status of the patients remains unaware: “*Yes, it actually happens so automated that you perhaps do it without knowing it*” [i4]. One nurse mentioned that the completion of the frailty screening increases her workload: “*Screening for frailty provides a structure for our daily activities. But because it has to* (it is mandatory, authors)*, it gives an increased workload, because you have to finish everything for the end of the shift”* [i1].

#### 1.4: prerequisites and suggestions for improvement

Nearly none of the nurses received a specific instruction at the time when the frailty screening tool was implemented in daily practice. Only one nurse mentioned that she received information on the screening during a more general geriatric training. Four nurses stated explicitly the need for training and instructions; this would improve the quality of the screening and follow-up in their view. In contrast, not all nurses mentioned this; only one nurse mentioned that the screening was introduced via a meeting years ago. This in contrast to the information of the geriatric nurse practitioners, in all hospitals the screening was introduced via courses, meetings or e-learnings. In addition, almost all nurses stated that an automatically generated alert in the digital nursing file via a pop-up would be helpful*. “I would prefer that the score is communicated via a pop-up notification in our digital nursing system, which you can’t ignore. You have to take action and then the alert stops”* [i7]. Some nurses thought that a sort of traffic light (‘alarm light’) in the patient file could also be helpful.

### Experiences and impact in daily practice (subthemes 2.5 and 2.6)

In all three hospitals, a systematic screening for frailty at admission had been implemented for more than 2 years and protocols were available regarding its conduct. Not all nurses were aware of this protocol though. One nurse stated: “*There is a protocol in our quality portal about this topic, but unfortunately this is only used in the other hospital site of our organization, but I think that would also be ideal for our site*” [i6]. Half of the nurses mentioned that the screening was conducted according to protocol. Although the screening results are automatically generated in the electronic patient file, not all nurses did know the screening results or could find the reported screening results in the digital file. One nurse mentioned: “*It is only a few mouse clicks on the computer, but it is not done at our ward*” [i8]. Nurses in one hospital stated that when a patient was screened as frail, this was not reported in the patient file.

#### 2.6 Briefing and knowledge

Nurses in one hospital stated that the screening results were not used during briefings between nursing shifts or transfers of patients between wards. These results were not checked before the briefing and the score itself was no topic during the briefing. The nurses in the other two hospitals reported that they used information about the frailty status only occasionally in their briefing between shifts and wards. Then it mostly was about specific items of the screening tools. *“If someone has fallen or has an increased risk of falling, that is sometimes used during briefings. Or if the patient was known with delirium last night, things like that are passed on, yes”* [i4]*.*

Only one of the nurses could mention the name of the frailty screening tool, as well as the cut-off score that is used to classify patients as frail. One nurse stated: *“I think that there is a bit of a knowledge gap on the screening tool. It is unclear what the screening score could mean for me as a nurse. But on the other hand, we generally know what to do in the next steps”* [i7]*.* Despite the daily use of the screening tools, nurses were not always aware of the screening. *“We use it* (frailty screening; author) *daily, but we were unaware about it” *[i6]*.* One nurse stated: *“I do not necessarily think that this is a lack of knowledge. I think we all know how to deal with frail older people and what we should do and ehmm ... I think it's more the awareness“* [i7].

#### 2.6 Actions after screening

Based on the cut off score of the frailty screening, consultation of a specialized geriatric team is available in all three included hospitals. In two hospitals, an order for consultation is automatically generated by the electronic nursing system based on the frailty sum score or on scores on specific items (i.e., in case of delirium or fall risk). On one ward, a geriatric consultant (nurse) performs consultation rounds based on the screening results: “*Every morning on weekdays there is a round of the geriatric nurse who visits patients with a high screening score”* [i1]. In some cases, an additional comprehensive assessment is used as follow-up. All nurses mentioned the Delirium Observation Scoring Scale (DOSS) [15] as a routine follow-up after a positive score on the delirium items of the frailty screening. Further assessments are also available for risk items such as malnutrition and functional decline. Almost all nurses mentioned that the outcome of the frailty score was presented in the daily medical consultation rounds, despite the daily nursing briefings. In one hospital, a tailor-made care plan based on the screening (conducted by a geriatric nurse) is used at the transfer between nursing wards (e.g., from acute admission unit to regular ward). In the other hospitals, a multi-disciplinary care plan was developed by ward nurses themselves, based on the score on different items of the screening tool (not the summative frailty score). Multidisciplinary interventions based on the outcomes of the frailty screening were reported, such as consultation of a physiotherapist, a dietician, activity teams and social service (transfer nurse). In all three hospitals, one nurse stated that family participation was encouraged in the case of frailty, to assist the patient during hospitalization. All nurses mentioned potential environmental actions if screening scores these warranted, such as transfer to a single room.

## Discussion

The aim of this exploratory study was to examine nurses’ experiences with and opinions about frailty screening at hospital admission and how this screening impacts their daily work. The participating nurses report that this screening can be useful to structure their work, create more awareness of frail older patients, serve as a starting point for pro-active nursing care and could encourage interdisciplinary collaboration in complex care for these patients. At the same time, they assess their clinical view as more important than the results of a standard tool, and the automatically generated recommendations based on these results may interrupt their ‘clinical alertness’. The nurses barely used the sum score of the screening but were particularly interested in the information from separate items of the screening, such as delirium or fall risk. Screening results are only partly embedded systematically in daily nursing care, e.g., in team briefings or during transfer of patients to other wards. Screening results on item level are used for the development of tailor-made care plans for frail older patients. The majority of the nurses received hardly any of training during implementation about the backgrounds of frailty screening and the use of the tools themselves.

The participants mentioned that frailty screening tools could be helpful for nurses, but neither these tools nor their summative scores were used during daily routines such as patient briefings or medical rounds. Nurses were more interested in the item scores of the tools. Perhaps this use of the screening tools is not that bad as the predictive power of summative scores is not convincing yet [21]. Focus on the item level seems to help nurses to structure their work and create awareness of frailty. Another explanation could be that misuse of the screening tools is due to the nurses’ lack of understanding about frailty in general and its implications for patients and care. Nurses mentioned their commonly known aspects of frailty at admission (i.e. delirium or falls), but seem to have less attention for other aspects or domains and the interaction between these domains.

It seems that the summative score and proposed cut-off points of the tools are probably more important for specialized geriatric teams, such as for case finding and related pro-active follow-up geriatric consultation.

Participants assessed their clinical view as more important than the results of standard tools, although these tools are considered helpful for less experienced nurses. These findings are partly in line with those of Hosie and colleagues who studied the perceptions of nurses with delirium screening in palliative care [23]. In contrast to our study, the nurses in Hosie’s study reported that, due to early screening, safety interventions were started pro-actively at an early stage. The screening tool was therefore helpful for them. Similar to our study, nurses also expressed their need for more tailored guidance and training in using screening tools. In Hosie et al.’s study, experienced nurses reported that the screening was not needed by ‘highly qualified nurses’. This is also consistent with our findings. Coker and colleagues studied frailty screening in multidisciplinary teams working in the community. They also concluded that education on frailty screening and training in the use of screening tools are necessary. All participants in their study expressed a desire for more training on frailty and the use of frailty screening tools [22].

The implementation of frailty screening needs careful consideration. Nurses stated that there was no training program at the time the frailty screening was implemented in the hospital. In our opinion, this has to be taken in account when implementing screening tools in daily practice. And in this implementation, educations should play a major role. Education has to be repeated every year and has to be an issue during structural peer reviews between nurses in general. Nurses need to learn about the relevance of frailty screening for their daily work. Education and training have to be delivered via different channels. In a study on frailty screening in nephrology services, the relevance of frailty screening was communicated via departmental presentations and ad-hoc one-on-one sessions. Animated videos on the purpose of frailty screening were displayed on TV screens in the department [28]. Educational interventions should not only focus on the nursing staff, but also on physicians, as they have the final say about patient treatment. Finally, unit leaders in hospitals should encourage the implementation of the learned material for optimal results [29].

Dedicated nurses could also take a part in this training. For instance, ward champions could be helpful in the implementation of screening and quality assurance on the nursing ward. Hospital specialized geriatric teams could take a part in coaching those dedicated nurses or champions in providing best evidenced care for frail older patients. Lim et al. suggest that the main stakeholders, including the multidisciplinary team of healthcare professionals, patients and their caregivers, should be involved from the time of hospital admission. Early involvement of the multi-disciplinary team in the practice of routine frailty screening in acute care settings will improve collaboration and communication in sharing essential patient information to develop holistic patient care goals [30].

Finally, digital nursing files could be more helpful for nurses. The results of the frailty screening were difficult to find in the files. Nurses could be more supported by the use of pop-ups or automatically generated alerts in case of frailty.

### Methodological reflection

This study is, as far as we know, the first one reporting on nurses’ opinions and experiences with frailty screening in hospital. We used an exploratory approach, gathering information in a variety of hospital settings among nurses from both internal and surgery wards. A limitation of this study is that, due to practical issues, not all interviews could be conducted in small groups as planned (in one hospital, individual nurses were interviewed instead). On the other hand a small sample size is a limitation. We think, however, that this variation had no large impact on our findings.

### Implications

It would be helpful when digital nursing files or systems were more supportive for nurses. Many data for frailty screening are already available in the digital system and this data could be automatically used in creating the frailty screening. In addition, alerts or popups could be helpful for nurses to create more alertness regarding frailty and useful for improving the quality of care.

In the hospitals that were included in our study, a systematic screening for frailty at admission was implemented for more than two years and protocols were available as to how the screening has to be executed. However, not all nurses were aware of this protocol. It is unclear from our results whether and how nurses were involved in the implementation process. In this process, it is necessary to claim time for training and education.; nurses need to learn why they have to screen older patients for frailty and what the benefits of screening are in their own daily work.

Further research is needed to gain more insight into the implementation of screening tools, including how the screening is conducted by nurses. Also, a more quantitative approach is needed to study the impact of screening in daily geriatric care.

## Conclusion

Most nurses stated that frailty screening tools are helpful in daily practice. However, nurses did not use the frailty screening tools in the referred way; tools were particularly used to evaluate patients on separate items of the tool instead of the summative score of the tool. When frailty screening tools are implemented in daily practice, training needs to be a focus. Additional research in this field is necessary to gain more insight in nurses’ opinions on frailty screening.

## Supplementary Information

**Additional file 1.**

## Data Availability

The datasets used and/or analysed during the current study are available from the corresponding author on reasonable request.

## Abbreviations

MFST-HP: Maastricht Frailty Screening Tool for Hospitalized Patients
GFI: Groningen Frailty Screening Index
VMS: Dutch National Safety Management Program for the screening of frail hospitalized older patients (Dutch abbreviated)
DOSS: Delirium Observation Scoring Scale
