# Supplementary material for: The opinions and experiences of nurses on frailty screening among older hospitalized patients. An exploratory study
Source: BMC Geriatr. 2021 Nov 3;21:624. doi: 10.1186/s12877-021-02586-z (PMC8565044; doi:10.1186/s12877-021-02586-z)
Supplement: Supplementary file 1 — Additional file 1. [file 12877_2021_2586_MOESM1_ESM.docx]

Interview guide

Introduction:

- Start-up
- Introduction of persons
- Purpose of study
- Duration of interview
- Confidentiality and anonymity
- Use of recordings
- Information about member checking

Start of the interview (record on).

This interview is about frail elderly patients and focuses on the use of frailty screening tools in a hospital setting. In your hospital, a screening tool for frailty is used at admission to map vulnerability in older patients. We are interested in your experiences with this tool.

1) About the screening and screening tool:

- What do you in general think of screening for frailty in older patients admitted to your hospital?
- How is screening executed in your hospital? What is your target population?
- What happens with the outcome of frailty screening?
- How does frailty screening get attention during the doctor’s rounds?
- How was the screening tool explained to you? Did you get any instructions on using the tool?
- What role does screening play within the nursing care process?
- How is the score interpreted?
- How is the geriatric consultation team involved?
- How is screening performed when a patient is known to be in cognitive decline?

2) Impact of the use of the screening instrument on daily nursing practice:

- In what way is frailty screening helpful in the daily care for older patients?
- Do you think frailty screening offers you a structure your work? If yes/no, why?
- What would you change in the current frailty screening tool or the screening process to improve the quality?
- Can you tell something about screening versus work pressure? How much time does it take to perform screening? What do you get in return?

Help/in-depth questions

• Can you explain that further for me?

• Can you give me an example of what you mean?

• Can you say something more about it?

• I don't quite understand ...

3) Are there any important issues that you would like to address in the context of screening for frail older patients that have not been discussed during this interview?

4) End of the interview. Recording stop. Hand out form for epidemiological data.

Thank you for your participation, openness and thinking about this subject. The results of our study will be forwarded to you later on via email. Please check it for factual inaccuracies.
